# Rectal Shaving in Colorectal Endometriosis Surgery—Definition, Evidence and Variability

**DOI:** 10.3390/jcm15176709

**Published:** 2026-08-29

**Authors:** Petya Tanovska, Jörg Keckstein, Mihai Constantin, Maya Sophie de Wilde, Petra Klein, Harald Krentel

**Affiliations:** 1 Department of Gynecology and Obstetrics, Klinikum Aschaffenburg-Alzenau, 63739 Aschaffenburg, Germany; petya.tanovska@klinikum-ab-alz.de (P.T.);; 2 Endometriosis Clinic Dres. Keckstein, 9500 Villach, Austria; 3 Carl-von-Ossietzky-University Oldenburg, 26129 Oldenburg, Germany; 4 Faculty of Medicine, Universidad Peruana Cayetano Heredia, Av. Honorio Delgado 430. San Martin de Porres, Lima 15000, Peru

**Keywords:** rectal shaving, deep infiltrating endometriosis, rectal endometriosis

## Abstract

**Objectives:** We aim to evaluate how rectal shaving is defined in the current literature, how consistently this definition is used, and what this means for the results and comparability of the respective research about surgery in deep rectal endometriosis. **Methods:** A literature search was performed in PubMed covering the period from 1990 to 2026, using multiple search terms combining “rectal shaving”, “shaving”, “conservative surgery” and “endometriosis”. Records were screened by title and abstract; methods sections of eligible publications were examined in full text, where accessible, to assess whether an explicit, anatomically anchored definition of rectal shaving (depth of excision, mucosal status, and bowel lumen involvement) was provided, benchmarked against the 2021 international consensus terminology. Information on definitions of rectal shaving, recurrence, and complication outcomes was extracted and compared. **Outcomes:** The review focused on how rectal shaving is defined, how variable the definition is used, and what this means for comparability of study results on recurrence and complication rates reported in the literature. **Results:** In studies using the term rectal shaving, the definitions of what rectal shaving means varied widely, ranging from superficial removal of serosa to full-thickness excision of the rectal muscular layer and the opening of the bowel lumen. This heterogeneity makes direct comparison of published literature difficult. Only a minority of publications provided a sufficiently explicit, anatomically anchored definition. Recurrence rates seem to be higher in patients with superficial excision compared to more radical resection, although this comparison is based on heterogeneous, predominantly observational data with inconsistent recurrence definitions, whereas complication rates are consistently lower in less radical surgeries. **Conclusions:** Definitions of rectal shaving in deep rectal endometriosis surgery are inconsistent in the available literature. This limits comparability of results regarding complication and recurrence rates. Future studies should clearly differentiate between superficial and deep excision of the muscularis layer, specifying whether mucosal integrity is preserved and whether suturing is required, and should explicitly reference standardized consensus terminology where available.

## 1. Introduction

Deep infiltrating endometriosis of the rectum is a challenging condition, often causing dyschezia, pelvic pain, diarrhoea, constipation, bloating and reduced quality of life [1,2]. Surgical treatment is frequently required when symptoms persist despite medical therapy, and several operative strategies have been described, ranging from laparoscopic rectal shaving to transanal discoid rectal resection and segmental rectal resection with anastomosis [3]. Among these approaches, rectal shaving is widely performed, particularly as part of a conservative, symptom-guided surgical philosophy aiming to preserve bowel integrity whenever possible. An international consensus-based terminology defined shaving as a partial thickness excision, without entering the lumen but requiring suture of the bowel. Lesions located on the peritoneal surface (serosa) of the rectum are considered peritoneal endometriosis and not bowel endometriosis [4]. However, the term “rectal shaving” is not used consistently across the literature, and different types of endometriosis excision on the rectal wall are referred to as “rectal shaving”, despite marked differences in surgical depth and anatomical involvement of rectal wall layers. For clarity, this review uses “rectal shaving” for partial-thickness excision that preserves the mucosa, in line with the 2021 consensus terminology [4]. “Conservative surgery” is used as an umbrella term covering both shaving and disc excision, as opposed to segmental resection. Several publications on laparoscopic management of rectal endometriosis illustrate the variability in using the term “rectal shaving”. In a large series reported by Donnez et al. [5], rectal shaving was described as meticulous excision of fibrotic endometriotic tissue from the anterior rectal wall during surgery for rectovaginal disease. The authors did not clearly differentiate between serosal, muscular, or mucosal layer involvement, but describe the technique as detachment of rectal endometriotic nodules from the anterior rectal wall to reach the correct cleavage plane. Similarly, another study by Reich et al., addressing laparoscopic treatment of cul-de-sac obliteration secondary to deep fibrotic endometriosis, reported shaving as a technique applied to the rectum during restoration of the pouch of Douglas, in which excision frequently extended into the muscularis propria while deliberately avoiding opening of the rectal lumen. In both studies, the term “shaving” encompassed a wide spectrum of surgical procedures, ranging from superficial serosal excision to deep muscular dissection, without standardized anatomical or surgical criteria [6].

Roman and colleagues used a more reproducible definition. In a long-term functional outcome study, rectal shaving was described as a bowel-preserving procedure for rectal endometriosis, in contrast to colorectal resection [7]. In a dedicated series, Roman et al. described rectal shaving as complete excision of rectal deep endometriosis while preserving the rectal mucosa and avoiding intentional opening of the rectal lumen [8]. This definition clearly separates shaving from disc excision and segmental colorectal resection. In a comprehensive video article, Chou et al. demonstrated the technique as “shaving” the lesion off the mucosa, ideally without entering the bowel lumen [9]. In contrast, other authors use the term rectal shaving for the description of superficial removal of endometriotic tissue confined to the rectal serosa or include the opening of the lumen as a possible part of rectal shaving [10,11].

In summary, the same term includes superficial excision, deep muscular excision, and sometimes opening of the bowel lumen in the existing literature and in daily practice. This lack of standardized definition leads to poor comparability of clinical results, heterogeneity in complication rates, and represents a bias in the interpretation of recurrence rates. A clearer understanding of how rectal shaving is defined and applied in the literature is therefore essential to improve comparability of future study results.

## 2. Materials and Methods

**Figure 1:**
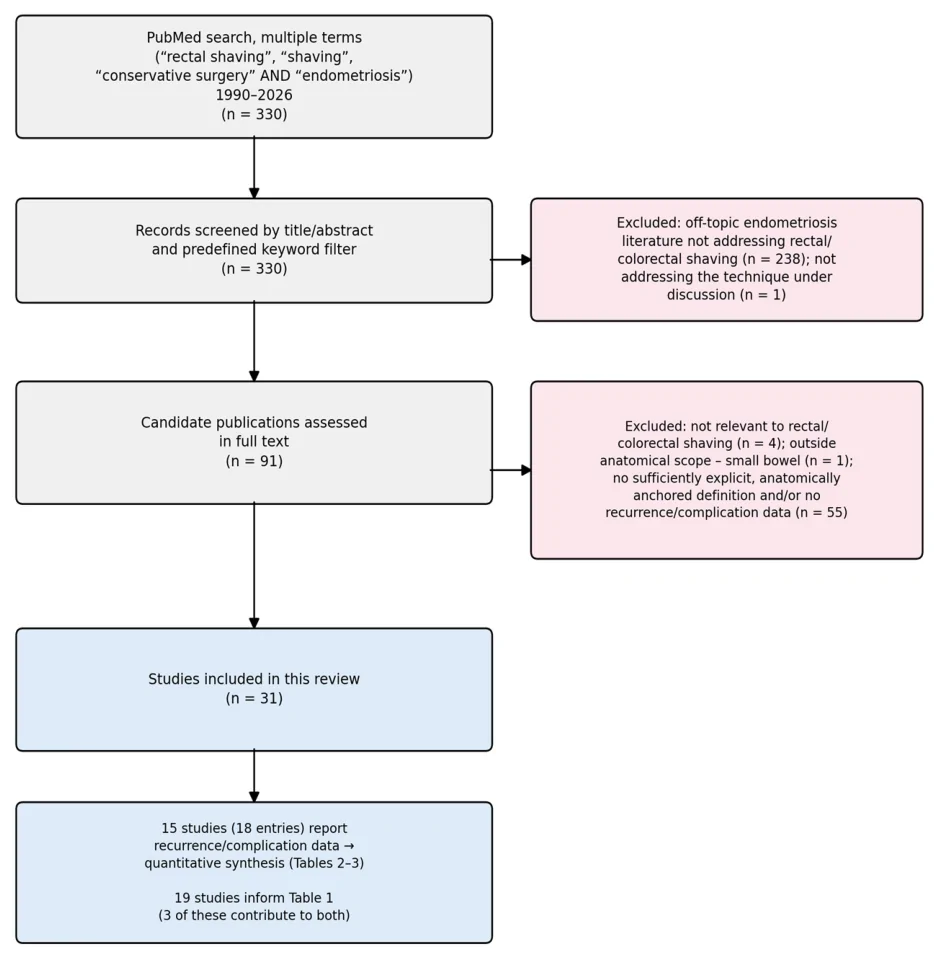
Flow diagram of study selection.

A structured literature search was conducted using PubMed, covering the period from 1990 to 2026. The two earliest studies included in this review, describing rectal shaving before the term itself became established in indexed publications (Reich et al., 1991 [6]; Donnez et al., 1995 [5]), fall within this search window and are discussed as original descriptive reports. The search combined the terms “rectal shaving”, “shaving”, “conservative surgery” and “endometriosis”, applied to titles and abstracts, with no language restriction, and was performed directly through the PubMed web interface; all records were exported and screened for title/abstract relevance using a predefined set of anatomical and surgical keywords (rectal, rectovaginal, bowel, colorectal, rectum, shaving, disc excision, segmental resection, nodular resection). Both prospective and retrospective surgical studies describing rectal shaving for deep infiltrating endometriosis were considered for inclusion. Eligibility was based on the following criteria: (1) involvement of rectal or colorectal endometriosis, (2) a description or definition of rectal shaving, and (3) reporting of recurrence and/or intraoperative and/or postoperative complications; publications providing only a definition of rectal shaving without recurrence or complication data were retained separately to inform Table 1 and the Discussion, but are not counted among the formally included studies shown in Figure 1. Studies focusing exclusively on diagnostic imaging, on endometriosis at sites other than the rectum/colon, or lacking sufficient surgical detail were excluded. For all eligible publications, the methods section was examined to determine whether an explicit, anatomically anchored definition of rectal shaving (depth of excision, mucosal status, bowel lumen involvement) was provided. This review is based on a single database (PubMed); complementary searches of other databases (e.g., Embase, Cochrane Library) were not performed.

Data extracted from eligible publications included definitions and terminology of rectal shaving, study design, recurrence rates, complication rates, and duration of follow-up. Results were summarised narratively and presented in tabular form to illustrate variability in definitions and reported outcomes.

The search retrieved 330 records (1990–2026). After title and abstract screening using the predefined keyword set described above, 239 records were excluded—238 as addressing endometriosis topics unrelated to rectal or colorectal shaving (e.g., ovarian endometrioma, hormonal therapy, presacral neurectomy), and one (a retrospective evaluation of intraoperative proctosigmoidoscopy) as not addressing the technique under discussion—leaving 91 candidate publications assessed in full text. Of these, four were excluded as not relevant to rectal/colorectal shaving, one was excluded as addressing small-bowel rather than rectal/colorectal disease, and 55 were excluded for lacking a sufficiently explicit, anatomically anchored definition of rectal shaving and/or recurrence/complication data, leaving 31 included studies (Figure 1). Of these, 19 inform the definition-evidence synthesis, and 15 report recurrence and/or complication data across 18 table entries in the quantitative synthesis; three studies contribute to both.

## 3. Results

### 3.1. Definition of Rectal Shaving

Across the included studies, substantial heterogeneity was observed in the definition of rectal shaving, and depth of excision is not consistently defined in the methods section of the available literature. Early reports from the 1990s used the term broadly for bowel-preserving dissection of rectovaginal or rectal disease, often without a clear layer-based definition [5,6]. In later clinical series, shaving continued to include deep muscular excision, while preservation of the bowel lumen remained an important but not uniformly applied feature [11]. In 2016, Roman et al. provided a more reproducible anatomical definition, describing rectal shaving as complete excision of rectal endometriosis with preservation of the mucosa and without intentional opening of the bowel lumen [8]. In 2021, the international consensus by Tomassetti et al. defined shaving as partial-thickness excision without intentional opening of the bowel lumen [4]. While in 2019, Khazali et al. reported rectal shaving as part of complete excision of deep endometriosis, without a clear layer-based definition [12], in 2024 the same group referred to the consensus statement in the description of the SOSURE approach in deep endometriosis surgery and thus adopted a standardized definition of terminology [13]. The historical development shows a transition from a broadly applied bowel-preserving surgical concept toward a more anatomically defined, mucosa-sparing technique (Table 1). Nowadays, although well-defined, the term rectal shaving continues to be used for a wide range of different surgical interventions on the rectal wall, including techniques in which bowel opening may occur as a consequence of deep excision [10].

**Table 1:** Evolution of definition and terminology of rectal shaving in deep rectal endometriosis surgery.

Year	Study	Definition of Rectal Shaving
1991	Reich et al. [6]	Early use of the term for deep rectal dissection extending into the muscularis propria without intentional lumen opening.
1995	Donnez et al. [5]	Broad bowel-preserving excision of rectovaginal disease without a clear layer-based definition.
2010	Donnez & Squifflet et al. [11]	Large clinical series showing that shaving was applied to deep rectovaginal nodules and was not limited to superficial serosal excision.
2010	Maytham et al. [14]	Reported shaving as superficial/partial-thickness removal reserved for serosal deposits, without opening the bowel lumen—an early example of anatomically anchored terminology.
2015	Roman et al. [15]	Described shaving as the mucosa-preserving first step preceding full-thickness disc excision when needed, reinforcing the lumen-preserving concept.
2016	Roman et al. [7]	Bowel-preserving approach linked to functional outcomes.
2016	Roman et al. [8]	More reproducible anatomical definition with preservation of the mucosa and avoidance of intentional lumen opening.
2016	Laganà et al. [16]	Review explicitly contrasting shaving (“no deeper than the muscular layer, preserving the mucosa”) with full-thickness excision.
2019	Khazali et al. [12]	Reported rectal shaving as part of complete excision of deep endometriosis, without a clear layer-based definition.
2020	Chou et al. [17]	Video article describing shaving of the muscular layer off the mucosa, ideally without entering the bowel lumen—anatomically precise.
2021	Tomassetti et al. [4]	International consensus definition of shaving as partial-thickness excision without intentional lumen opening.
2021	Donnez O. et al. [10]	Broader concept of “deep shaving,” extending into the muscularis and potentially resulting in bowel opening.
2022	Xu et al. [18]	Described shaving as detachment of lesions without penetrating the intestinal cavity, sparing mucosa and deep muscle for lesions < 2 cm.
2023	Gaia et al. [19]	Robotic multi-site “mucosa skinning” explicitly sparing the intestinal mucosa.
2024	Khazali et al. [13]	Description of the SOSURE approach explicitly references the 2021 consensus terminology.
2025	Vallée et al. [20]	Meta-analysis defining shaving with preservation of the mucosa and a segment of the muscularis, while noting risk of unintentional lumen opening.
2025	Tsuei et al. [21]	Review using near-identical operational language to Vallée et al. [20].
2025	Zhang et al. [22]	Systematic review defining shaving as excision preserving mucosal integrity without entering the lumen.
2025	Collinet et al. [23]	Describes shaving performed “as deep as possible,” occasionally requiring full-thickness repair.

Note: Table 1 highlights the subset of included studies that specifically mention the definition/terminology of rectal shaving; three of these (Donnez & Squifflet [11], Roman et al. 2016 [8], and Collinet et al. [23]) also contribute recurrence or complication data.

To assess whether adoption of the 2021 consensus terminology has improved over time, the methods sections of all eligible publications introducing or reporting a definition of rectal shaving (Table 1) were examined for an explicit, anatomically anchored definition of rectal shaving, benchmarked against the Tomassetti et al. [4] terminology. Several of these publications predate the 2021 consensus and independently converge on a similar partial-thickness, mucosa-preserving, lumen-avoiding description, indicating that anatomically precise definitions of rectal shaving were achievable well before the formal consensus was published. Of the seven extracted publications published after 2021 that provided their own explicit operational definition, five (Xu et al., 2022 [18]; Gaia et al., 2023 [19]; Vallée et al., 2025 [20]; Tsuei et al., 2025 [21]; Zhang et al., 2025 [22]) describe the technique in terms broadly consistent with the consensus definition—partial-thickness excision with preservation of the mucosa—yet none explicitly cites the Tomassetti et al. [4] terminology as its source. Khazali et al. [13], in their 2024 description of the SOSURE approach, is the only identified post-2021 publication to explicitly reference the consensus terminology. Collinet et al. [23] described a shaving technique performed “as deep as possible into the thickness of the rectal wall”, occasionally requiring repair of a full-thickness defect.

### 3.2. Recurrence Rates

Publications specifically reporting recurrence after rectal shaving remain limited, and reported recurrence rates varied across the available studies (Table 2). In a retrospective study of 122 patients undergoing rectal shaving, Roman et al. reported an overall recurrence rate of 4.0%. The recurrent lesions were subsequently managed by repeat shaving in 0.8%, disc excision in 0.8%, and segmental resection in 2.4% of patients [8]. In a randomized trial and its long-term follow-up studies, recurrence after conservative surgery was reported in 3.7% of patients at 5 years and 7.4% at 7 years, compared with 0% after segmental colorectal resection at both follow-up points. However, the conservative group included both rectal shaving and disc excision and therefore does not provide a shaving-specific recurrence estimate [24,25,26]. At the 10-year follow-up of the same trial cohort, recurrence was reported in 7.4% after excision versus 3.6% after segmental resection (OR 0.46, 95% CI 0.04–5.4; *p* = 0.54) [27]. In a systematic review and meta-analysis, Bendifallah et al. also showed that recurrence was more frequent after shaving than after disc excision or segmental resection, with rates of 8.3%, 5.7%, and 2.7%, respectively, in a subgroup analysis restricted to histologically confirmed recurrence (i.e., the histology-confirmed comparative subset of four studies within this systematic review; the review’s overall pooled recurrence rate across all 41 included studies was 8.1% for shaving, 10.4% for disc excision, and 8.6% for segmental resection, as shown separately in Table 2). The pooled odds ratio was 5.53 for shaving versus segmental resection (95% CI 2.33–13.12; *p* = 0.001) and 3.83 for shaving versus disc excision (95% CI 1.33–11.05; *p* = 0.01) [28].

### 3.3. Complication Rates

Complication rates after rectal shaving are summarized in Table 3. Reported complications include both intraoperative adverse events (e.g., rectal perforation and ureteral injury occurring during dissection) and postoperative complications (e.g., fistula formation, bladder atony, anastomotic or bowel-wall stenosis); the two categories are distinguished where the source studies report them separately. In a prospective study by Donnez and Squifflet, major intraoperative and early complications after rectal shaving included rectal perforation in 1.4%, ureteral injury in 0.8%, blood loss > 300 mL in 0.2%, and urinary retention in 0.8% [11]. In the largest shaving series, including 3298 patients, Donnez et al. reported major complications such as rectal perforation in 1.3%, transient ureteral retention in 0.64%, ureteral injury in 0.3%, and fecal peritonitis in 0.04% [29]. In a rectal shaving cohort using plasma energy, Marty et al. observed no intraoperative complications [30]. Postoperatively, rectovaginal fistula occurred in 0.9%, rectouterine fistula in 0.9%, and bladder atony requiring ≥3 weeks of self-catheterization in 1.8%. Roman et al. reported Clavien–Dindo grade IIIa, IIIb, IVa, and IVb complications in 0.8%, 5.7%, 1.6%, and 0.8%, respectively, and rectal fistula formation in 1.6% [8].

Comparative studies generally show a higher burden of severe complications after segmental colorectal resection than after conservative rectal surgery. Roman et al. reported Clavien–Dindo grade III complications in 22.0% after conservative surgery and in 30.3% after segmental resection, whereas bowel stenosis requiring an additional procedure was observed only after segmental resection (0% vs. 15.2%) [24]. In the three-arm cohort published by Abo et al., Clavien–Dindo grade IIIb complications occurred in 11.8% overall, with two-thirds of these events occurring after segmental resection; rectovaginal fistula was reported in 2.1% after shaving, 3.7% after discoid excision, and 5.8% after segmental resection [3]. In a multicenter study, Hudelist et al. described grade III–IV complications in 3.7% after shaving, 2.7% after discoid excision, and 7.8% after segmental resection [31]. In a single-center cohort, Bafort et al. reported Clavien–Dindo grade III complications in 1.6% after conservative surgery and 10.5% after segmental resection, though the conservative group was not limited to rectal shaving alone [32]. Similarly, Hernández Gutiérrez et al. reported severe postoperative complications in 0% after shaving, 5.0% after discoid excision, and 23.5% after segmental resection [33].

**Table 3:** Key studies reporting complication rates after rectal shaving and comparative bowel surgery for deep endometriosis.

Study	Design/Population	Comparator Groups	Complication Outcomes	Interpretation
[11] Donnez & Squifflet, 2010	Prospective single-arm shaving series.	*n* = 500; rectal shaving only.	Rectal perforation 1.4% (7/500); ureteral injury 0.8% (4/500); blood loss >300 mL 0.2% (1/500); urinary retention 0.8% (4/500).	Dedicated shaving cohort; low major morbidity.
[29] Donnez et al., 2013	Large single-arm shaving series.	*n* = 3298; rectal shaving only.	Rectal perforation 1.3% (42/3298); transient ureteral retention 0.64% (21/3298); ureteral injury 0.3% (10/3298); fecal peritonitis 0.04% (1/3298).	Very large shaving experience with very low major complication rates.
[8] Roman et al., 2016	Retrospective single-arm shaving series.	*n* = 122; rectal shaving only.	Clavien-Dindo IIIa 0.8%; IIIb 5.7%; IVa 1.6%; IVb 0.8%; rectal fistula 1.6%.	Most relevant Roman study for complications after shaving alone.
[30] Marty et al., 2017	Retrospective plasma-energy shaving series.	*n* = 110; rectal shaving only.	No intraoperative complications. Rectovaginal fistula 0.9%; rectouterine fistula 0.9%; bladder atony ≥ 3 weeks 1.8%; Clavien-Dindo IIIa 0.9%, IIIb 4.5%, IVa 0.9%.	Low morbidity after plasma-energy shaving.
[24] Roman et al., 2018	Randomized trial.	Conservative surgery *n* = 27 vs. segmental resection *n* = 33.	Clavien-Dindo III 22.0% vs. 30.3%. Rectovaginal fistula 7.4% vs. 0%. Pelvic abscess 0% vs. 3.0%. Bowel stenosis requiring additional procedure 0% vs. 15.2%.	Conservative arm was not shaving alone.
[3] Abo et al., 2018	Three-arm comparative cohort.	Shaving *n* = 145; discoid excision *n* = 80; segmental resection *n* = 139.	Overall Clavien-Dindo IIIb 11.8%; two thirds after segmental resection (*p* < 0.001). Rectovaginal fistula 2.1% vs. 3.7% vs. 5.8%.	Severe morbidity concentrated in the segmental resection group.
[33] Hernández Gutiérrez et al., 2019	Comparative cohort.	Segmental resection *n* = 76; discoid excision *n* = 20; shaving *n* = 47.	Severe complications 23.5% vs. 5.0% vs. 0% (*p* = 0.005). Rectovaginal fistula 5.2% vs. 0% vs. 0%; permanent urinary retention 5.2% vs. 0% vs. 0%.	No severe postoperative complications after shaving in this cohort.
[32] Bafort et al., 2020	Single-center comparative cohort.	Conservative *n* = 61 vs. segmental resection *n* = 171.	Clavien-Dindo III 1.6% vs. 10.5%.	Conservative arm not limited to shaving alone.
[31] Hudelist et al., 2022	Retrospective multicenter comparative study.	Shaving *n* = 328; discoid excision *n* = 222; segmental resection *n* = 387.	Grade III–IV 3.7% vs. 2.7% vs. 7.8%. Anastomotic leakage 0.6% vs. 1.4% vs. 3.6%; fistula 0.9% vs. 0.5% vs. 1.6%.	Largest multicenter comparison; severe morbidity highest after segmental resection.
[34] Roman et al., 2020	Retrospective cohort (CIRENDO registry); *n* = 1102 patients with deep endometriosis of the rectosigmoid.	Shaving (31.9%) vs. disc excision (23.1%) vs. colorectal resection (35.8%) vs. combined disc + segmental resection (2.9%).	Overall bowel fistula 3.4% (37/1102). Rectal lumen opening increased fistula risk vs. shaving regardless of nodule size: adjusted OR 6.8 (95% CI 1.9–23.8) for disc excision, 4.8 (1.4–16.9) for colorectal resection, 11.0 (2.1–58.6) for combined disc + segmental resection.	Largest available comparative dataset directly linking bowel lumen opening (rather than the technique label itself) to increased fistula risk, supporting the anatomical rationale for mucosa/lumen-preserving shaving.
[23] Collinet et al., 2025	Retrospective single-center cohort; *n* = 97 patients treated for colorectal endometriosis.	Segmental resection (*n* = 43), single-disc excision (*n* = 20), double-disc excision (*n* = 5), rectal shaving (*n* = 29); complications not stratified by technique.	Overall complications 14%; severe complications (Clavien–Dindo ≥ 3) 8.24%; rectovaginal fistula 3.09%.	Complication rates were not reported separately for shaving.

## 4. Discussion

Although in 2021 an international consensus defined rectal shaving as partial-thickness excision with preservation of the mucosa and without intentional opening of the lumen [4], a universally accepted definition of what rectal shaving exactly means is still lacking, and different interpretations continue to be used in the literature and in daily practice. The same term is used for procedures with different surgical techniques, including superficial excision, deep mucosa-sparing excision, and full-thickness excision including intentional opening of the bowel lumen. It is noteworthy that the concept of rectal shaving has developed over time. Initially, the authors described surgery for rectovaginal disease as complete excision of nodules, without clear differentiation between the applied type of excision and excision depth [5,6]. These earlier studies show that the term “shaving” was initially used for a broader surgical concept rather than for a strictly defined anatomical technique. In a later prospective series, rectal shaving was still presented as a conservative surgical approach, however related to a rectal perforation rate of 1.4%, indicating that excision was not limited to superficial layers [11].

In recent reviews, Donnez and Donnez state that shaving is feasible independently of lesion size and feasible in >95% of deep rectal endometriosis cases, and describe shaving as dissection along the rectal muscularis, with bowel opening as a consequence of deep shaving [10,35]. This definition extends shaving toward deeper excision and overlaps with full-thickness techniques like discoid excision. In contrast, Roman et al. provide a more reproducible definition, in line with the international consensus, describing rectal shaving as excision of disease from the serosa and/or muscularis with preservation of the mucosa and avoidance of lumen opening. This definition excludes incomplete excision and distinguishes rectal shaving clearly from disc excision and segmental colorectal resection [8]. In their most recent 10-year follow-up analysis of this trial cohort, Roman et al. use “nodulectomy” as an umbrella term encompassing both shaving and disc excision, as opposed to segmental resection [27]—a terminological convention that parallels the conservative-surgery/segmental-resection distinction adopted in this review. Other authors define shaving by what it is not, when compared to disc excision and segmental resection. In most of the existing literature, a clear definition of what exactly is meant by “rectal shaving” is missing from the methods section; this also extends to large, frequently cited series that are not specifically framed as shaving studies. Bourdel et al. included patients with at least invasion of the serosa of the rectum in rectovaginal nodules exceeding 2 cm in a comparative study of different techniques in the management of rectovaginal endometriosis [36]. Since serosal disease is not considered rectal endometriosis and is not equivalent to nodules infiltrating the muscularis layer, this illustrates that the criteria used to identify a “rectal” nodule requiring surgery are inconsistently anchored to a specific anatomical depth. This pattern is not confined to older literature: as detailed in Section 3.1, the seven identified post-2021 publications providing their own surgical definition of rectal shaving describe a technique broadly consistent with the consensus definition in substance, yet do not specify infiltration depths, nodule size, and whether suture is needed or not. Khazali et al. [13] is the only identified publication to explicitly reference the consensus by Tomassetti et al. [4]. Taken together, these findings suggest that the definitional substance of the 2021 consensus has been adopted in the literature, but also show that explicit citation of the consensus terminology as its source remains rare, and that the heterogeneity in how rectal shaving is used and reported is not a historical discussion predating standardization efforts, but a persistent feature of current publication practice. In our clinical experience, this inconsistent adoption is not confined to the published literature reviewed here: the same imprecise, non-anatomically anchored use of the term is present in conference presentations, case discussions, and everyday clinical decision-making, where “shaving” is often used as a general label for conservative, bowel-preserving surgery without further specification of excision depth. Taken together, these findings show that the term rectal shaving is used for fundamentally different procedures with differences in complication risks and recurrence rates. This likely explains the variability in reported recurrence and complication rates across studies. In summary, rectal shaving is associated with lower rates of major postoperative complications compared with disc excision or segmental colorectal resection [3]. Long-term functional outcomes, particularly regarding bowel function, appear to be more favorable after rectal shaving than after radical resection, while the risk of recurrence following rectal shaving, especially in cases of deep rectal infiltration, seems to be higher compared to disc resection and segmental resection [7]. However, recurrence estimates should be interpreted with caution given that several comparative studies combine shaving with other conservative procedures rather than reporting a shaving-specific result. As recurrence rate seems to be related to the completeness of excision of the disease and thus the depth of excision, a meaningful description of the procedure in the method section of further studies should specify the depth of excision, the resulting residual wall thickness, and whether fibrotic or endometriotic tissue was deliberately retained to maintain bowel-wall integrity, and thus completeness of excision of the nodule was achieved or not.

Modern imaging enables preoperative characterization of lesion size and bowel-wall infiltration, thereby supporting classification-based surgical planning. Based on presurgical imaging and the #Enzian classification, we recently described complete nodular resection (CNR), a mucosa-sparing complete excision of the rectal nodule and surrounding muscular layer with a single-layer transversal suture adopting the rectal wall [37,38]. A comparable technique using laparoscopic submucosal liquid injection to facilitate separation of the muscularis from the mucosa was described by Chou et al. [9]. Intraoperative transvaginal, transrectal, or laparoscopic ultrasound may additionally help verify complete lesion excision [39,40], and a recent feasibility study specifically evaluated multifaceted intraoperative ultrasound to facilitate the shaving technique itself [41], while standardized early postoperative imaging could document the immediate anatomical result and provide a reference for subsequent follow-up. Without such documentation, persistent disease deliberately or inadvertently left behind may be misclassified as recurrence. Interpretation is further complicated by postoperative oedema, fibrosis, and wall duplication following shaving and suturing, which may mimic residual disease. Moreover, comparisons with discoid or segmental resection are affected by selection bias, as shaving is generally performed in smaller or technically more favorable lesions, whereas larger, multifocal, stenotic, or deeply infiltrating lesions are more often treated by bowel resection. Outcomes also remain highly operator-dependent because shaving is not a uniform procedure, and published studies use heterogeneous definitions of recurrence, ranging from recurrent symptoms or imaging abnormalities to histologically confirmed disease and reoperation.

This review has several limitations. The search was restricted to a single database (PubMed) using multiple terms, and a narrative rather than a fully systematic multi-database synthesis was performed; no complementary databases (e.g., Embase, Cochrane Library) were searched. Reliance on title/abstract indexing may still have missed relevant studies that describe comparable procedures using different terminology; this risk is partly mitigated by discussion of additional relevant publications identified through targeted searching. No formal risk-of-bias or quality-appraisal tool (e.g., ROBINS-I, AMSTAR-2) was applied across the heterogeneous mix of single-center cohorts, randomized trials, and systematic reviews/meta-analyses summarized in Table 2 and Table 3. The available evidence on rectal shaving is predominantly observational and heterogeneous in design, recurrence definition, and follow-up duration, which limits the strength of any conclusions about the comparative effectiveness of shaving versus disc excision or segmental resection.

Future research, especially when comparing different surgical techniques in the management of symptomatic deep rectal endometriosis, should be based on a standardized definition of what rectal shaving exactly means. This approach would allow for better comparability of outcomes, such as complication and recurrence rates, and help overcome the uncertainty related to the term “rectal shaving”. Meaningful comparison of rectal shaving with discoid or segmental resection will require prospective studies using standardized indications, a reproducible description of the achieved excision depth, early postoperative reference imaging, and separate reporting of residual disease.

## 5. Conclusions

Rectal shaving is a widely used conservative surgical technique for the treatment of rectal deep infiltrating endometriosis. However, there is substantial variability in how rectal shaving is defined and performed. This limits direct comparison of recurrence and complication outcomes. While the 2021 international consensus established a formal definition of rectal shaving, the present review shows that this definition has not yet been consistently adopted in practice. A global guideline on diagnosis and treatment of rectal endometriosis, including standardized intraoperative and early postoperative imaging, would help to improve comparability of studies, support balanced, patient-centered surgical decision-making and to avoid incomplete excision and be essential to distinguish deliberately or inadvertently retained disease from true recurrence.

## Figures and Tables

**Table 2:** Key studies reporting recurrence after rectal shaving or conservative rectal surgery for deep endometriosis.

Study	Design/Population	Comparator Groups	Follow-Up	Recurrence Outcomes	Interpretation
[8] Roman et al., 2016	Retrospective series using prospectively recorded CIRENDO data; 122 consecutive patients undergoing rectal shaving for rectal DIE.	Primary procedure: rectal shaving.	1–6 years	Rectal recurrences occurred in 4.0% overall.	Most specific Roman study for shaving-related recurrence.
[24] Roman et al., 2018	Randomized trial; 60 enrolled (27 conservative surgery, 33 segmental resection).	Conservative surgery = shaving or disc excision; comparator = segmental colorectal resection.	24 months	Defined the trial arms but did not provide the later 5-/7-year recurrence estimates.	Clarifies that the conservative arm in later follow-up papers was a combined group, not shaving alone.
[25] Roman et al., 2019	Five-year follow-up of the randomized trial; 55 women analyzed.	Excision arm (shaving or disc excision) vs. segmental colorectal resection.	5 years	5-year recurrence: 3.7% after conservative excision vs. 0% after segmental resection.	Not a pure shaving recurrence rate; applies to the combined conservative arm.
[26] Roman et al., 2022	Seven-year follow-up of the randomized trial; 55 women analyzed.	Excision arm (shaving or disc excision) vs. segmental colorectal resection.	7 years	7-year recurrence (new DIE nodules infiltrating the rectum): 7.4% vs. 0%.	Combined conservative arm, not shaving-only.
[27] Roman et al., 2026	Ten-year follow-up of the randomized trial; 55 women enrolled, 5 lost to follow-up before 10 years (9.1%).	Excision arm (shaving or disc excision) vs. segmental colorectal resection.	10 years	10-year recurrence: 7.4% after excision vs. 3.6% after segmental resection (OR 0.46, 95% CI 0.04–5.4; *p* = 0.54); primary functional endpoint met in 74.1% vs. 71.4% (OR 0.88; *p* = 0.83); no significant differences in bowel-function, quality-of-life, or continence scores between arms.	Longest available follow-up of this trial; confirms no significant difference in recurrence or functional outcomes between conservative and radical rectal surgery at 10 years.
[28] Bendifallah et al., 2020 (full systematic review, all 41 studies)	Systematic review of 41 studies reporting recurrence after colorectal endometriosis surgery.	Shaving vs. disc excision vs. segmental resection.	12–108 months	Shaving 108/1339 (8.1%), disc excision 21/202 (10.4%), segmental resection 198/2304 (8.6%). No significant difference overall.	Descriptive context only; recurrence definitions heterogeneous across studies.
[28] Bendifallah et al., 2020 (histology-confirmed subset)	Meta-analysis of 4 comparative studies with histologically proven recurrence.	Shaving vs. disc excision vs. segmental resection.	Comparative studies only	Shaving 33/401 (8.3%), disc excision 6/106 (5.7%), segmental resection 8/300 (2.7%). OR shaving vs. segmental 5.53 (95% CI 2.33–13.12; *p* = 0.001); shaving vs. disc 3.83 (95% CI 1.33–11.05; *p* = 0.01).	Best comparative evidence that histology-proven recurrence is more frequent after shaving.

## Data Availability

All data supporting the reported results are available from the corresponding author on reasonable request.

## Abbreviations

The following abbreviations are used in this manuscript:

DIE	Deep infiltrating endometriosis
CNR	Complete nodular resection
